# Toward Practical Zn‐Ion Batteries Through Separator/Electrolyte Co‐Design

**DOI:** 10.1002/advs.78250

**Published:** 2026-10-29

**Authors:** Gao Wang, Ze He, Rui Wang, Jiajing Wang, Shaowen Cao, Michael De Volder, Qinyou An

**Affiliations:** ^1^ Key Laboratory of Advanced Technology for Materials Synthesis and Processing Wuhan University of Technology Wuhan China; ^2^ Institute For Manufacturing Department of Engineering University of Cambridge Cambridge UK

**Keywords:** electrolyte, energy storage, glass fiber, materials science, plating

## Abstract

While substantial research effort has been paid to address short‐circuits and parasitic hydrogen evolution reactions (HER) in Zn‐ion batteries (ZIBs), a remaining challenge that is often overlooked is ZIB separators. Currently, thick glass fiber (GF) separators are used, which unnecessarily reduce the volumetric cell performance and lead to non‐uniform Zn plating/stripping. Unfortunately, separators optimized for Li‐ion batteries are intrinsically hydrophobic and therefore incompatible with aqueous ZIB systems. Here, we propose using a hybrid organic‐aqueous electrolyte which is compatible with thin polypropylene (PP) separators developed for Li‐ion batteries. The hybrid electrolyte, together with the PP separator, enables uniform Zn plating and suppresses HER. Combined, these effects allow for stable cycling for 4360 h at 1 mA cm^−2^ and 3 mAh cm^−2^ in a Zn symmetrical cell. Further, replacing the currently used 350 µm‐thick GF separator with our proposed 23 µm PP separator can increase the energy densities of multi‐layer pouch cells by over 2.5‐fold. Finally, Zn//NVO full cells using our hybrid electrolyte and separator demonstrate stable cycling performance for over 200 cycles at a high areal capacity of 4.4 mAh cm^−2^, demonstrating that electrolyte/separator co‐design is an overlooked strategy toward practical ZIBs.

## Graphical abstract

A hybrid organic–aqueous electrolyte enables thin commercial polypropylene separators to replace bulky glass‐fiber separators in practical Zn‐ion batteries. This electrolyte/separator co‐design improves wettability, promotes uniform Zn plating/stripping, and suppresses hydrogen evolution, resulting in 4360 h of symmetric‐cell cycling and stable Zn//NVO full cell operation at 4.4 mAh cm^−2^ while increasing pouch‐cell energy density by over 2.5‐fold.

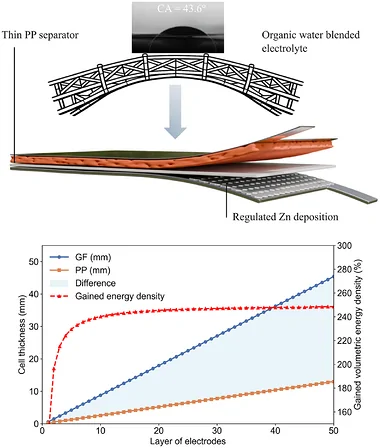

## 1 Introduction

The deployment of renewable energy is key to meeting the growing energy demands of human society while at the same time alleviating the challenges with emissions from fossil fuels [1, 2, 3]. However, the intermittency of renewable energies such as solar and wind is a bottleneck in their deployment, which necessitates the development of large‐scale energy storage systems (LSESSs) [4, 5, 6]. Although lithium‐ion batteries (LIBs) are an ideal solution for portable devices and vehicles, the limited Li resources and their relatively high cost and flammability unfortunately complicate their application in LSESSs [7, 8, 9]. Over the past decades, substantial efforts have been made to develop suitable LSESSs, with a focus on cost‐effectiveness, safety, and durability [10, 11]. Aqueous zinc ion batteries (ZIBs) have emerged as a promising candidate due to their abundant resources, low cost, and inherent safety [10, 12, 13].

Nevertheless, several critical challenges of ZIBs hinder their application. First, Zn metal anodes suffer from dendrite growth, corrosion, and the parasitic hydrogen evolution reaction (HER) in aqueous electrolytes, making it difficult to meet the cycle life required for LSESS [14, 15]. In addition, a challenge that is less studied so far is the lack of commercial separators suitable for ZIBs [16, 17, 18, 19, 20]. Most of the ZIBs reported so far use glass fiber (GF) filtration membranes as separators because battery separators optimized for LIBs are hydrophobic. Unfortunately, GF membranes (i) are thick (hundreds of micrometers), (ii) require large amounts of electrolyte to be filled, and (iii) have pores that are not optimized for battery operation. Overall, this results in a high portion of volume and mass of inactive components within the cell and suboptimal energy density [18, 21, 22]. As illustrated in Figure 1a and Figure S1, the commonly used GF separator (350 µm) is much thicker than cathode and anode electrodes (normally lower than 150 µm), whereas in LIBs commercial separators are usually less than 25 µm and thinner than the electrode coatings [23, 24, 25].

**FIGURE 1:**
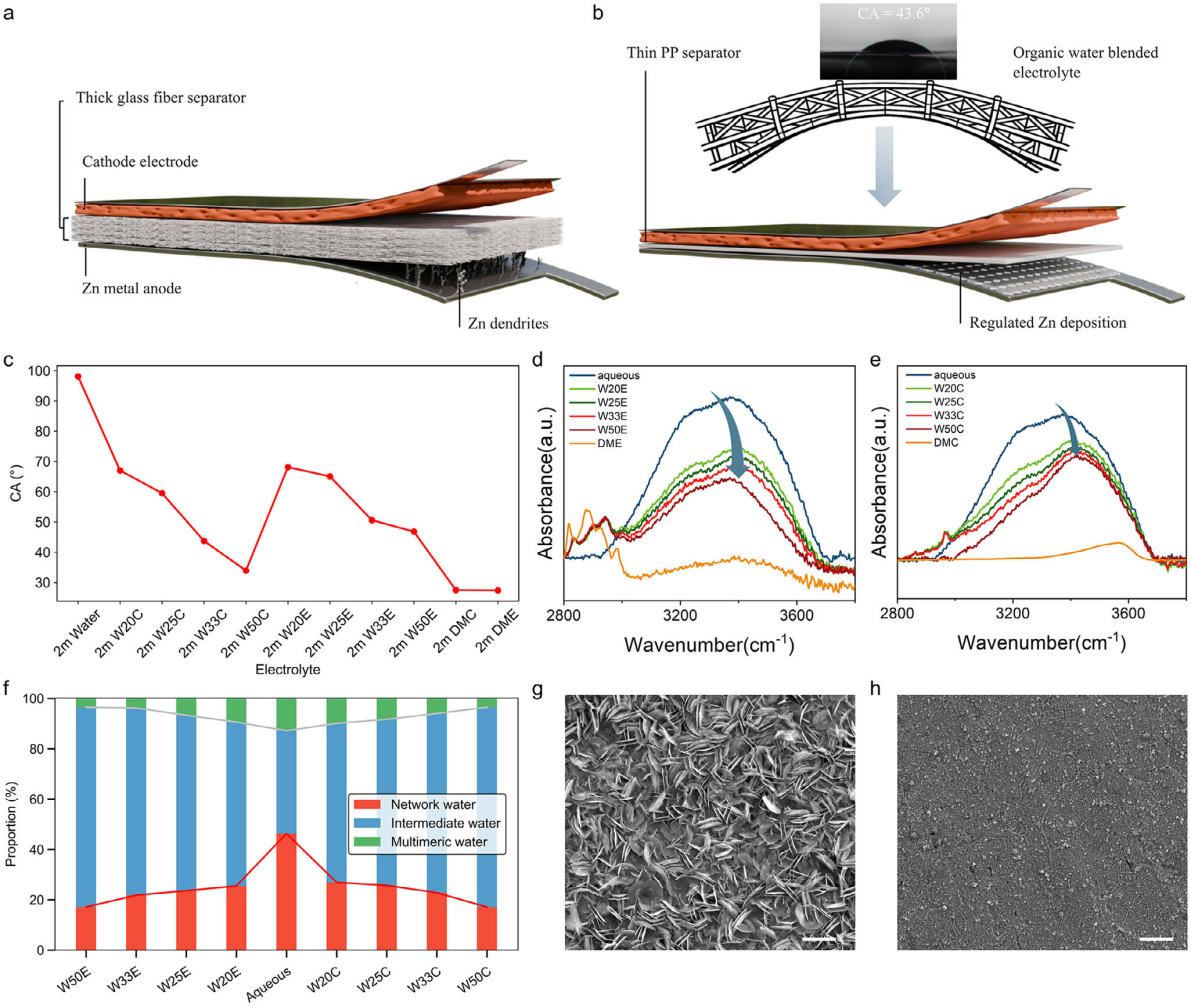
(a, b) Schematic illustration of (a) ZIBs using a GF separator with an aqueous electrolyte and (b) ZIBs with a PP separator and a hybrid organic‐aqueous electrolyte. (c) Contact angles of various electrolytes on the PP separator. (d, e) FTIR spectra of various electrolytes (W50C‐W20C, W50E‐W20E, and aqueous (2 mW)). (f) Proportions of network water, intermediate water, and multimeric water in various electrolytes (W50C‐W20C, W50E‐W20E, and aqueous (2 mW)). (g, h) SEM images of Zn foils after immersion in (g) the aqueous 2 mW electrolyte and (h) the W33C electrolyte for 24 h. Scale bars, 10 µm.

To address dendrite growth, corrosion, as well as the HER of Zn metal anodes, organic solvent/water blended electrolytes have been suggested as a promising strategy to balance the advantages of aqueous non‐flammable and highly conductive electrolytes with those of solvent's enhanced electrochemical stability. Wang et al. introduced dimethyl sulfoxide (DMSO) into ZIB electrolytes and demonstrated that DMSO can partially replace water molecules in the Zn^2+^ solvation shell, thereby suppressing HER [26]. Subsequently, a series of other organic solvents, including DME [27], ACN [28], Methanol [29], Glycerol [30], TEP [31], Sulfolane [32], etc., have been developed that can effectively modify Zn^2+^ solvation structure, regulate Zn deposition, and mitigate parasitic HER [33]. Regarding separator engineering, current efforts have predominantly focused on the modification of GF separators. Li et al. deposited vertical graphene carpet on the surface of GF separators, resulting in Janus separators with regulated electric field and Zn^2+^ flux [34]. Similarly, Hou et al. coated tin metal layers onto the surface of GF separators to regulate Zn deposition behavior [35]. Although these studies demonstrate improved cycling performance, they do not address the fundamental issue of excessively thick separators, which continues to compromise cell‐level energy density.

In this work, we address this issue by using a hybrid organic‐aqueous ZIB electrolyte that wets a thin commercial polypropylene (PP) separator (Celgard‐2500) and at the same time reduces HER. Previous studies mainly rely on cellulose/glass fiber separator modification or on electrolyte engineering, yet most modified separators involve complicated fabrication procedures and relatively large thicknesses [19, 20, 36, 37, 38]. Different from these strategies, our hybrid electrolytes avoid additional separator modification. Figure 1b depicts the volumetric gains in cell density by leveraging a thin PP separator developed for LIBs. The key to making this possible is to simultaneously optimize hybrid electrolytes for compatibility with the PP separator and for regulating Zn plating and reducing HER. We developed a series of hybrid organic‐aqueous electrolytes and investigated their compatibility with the PP separator and the Zn metal anode. Among them, the hybrid electrolyte containing 33 mol% DMC (denoted as W33C) delivers outstanding cycling stability, enabling stable operation for over 4300 h at a practical test areal capacity of 3 mAh cm^−2^. Post‐mortem scanning electron microscopy (SEM), transmission electron microscopy (TEM), x‐ray diffraction (XRD), and x‐ray photoelectron spectroscopy (XPS) analyses were performed to elucidate the synergistic effects of the hybrid electrolyte, the PP separator, and side reactions in achieving stable ZIB operation. Finally, we evaluate the combination of the W33C electrolyte and the PP separator in practical cell configurations under conditions of high mass loading (12.5 mg cm^−2^), relatively lean electrolyte (10 g Ah^−1^), and a low negative‐to‐positive capacity ratio (N/p = 2). The Zn//NVO full cell using the W33C electrolyte and the PP separator delivered a high areal capacity of 4.4 mAh cm^−2^ and maintained stable cycling for over 200 cycles.

## 2 Results and Discussion

The PP Celgard‐2500 separator has a dense, uniform, and interlaced micron‐scale porous network structure, and its uniformly distributed pores with a narrow size distribution ensure uniform ion transport and avoid local dry‐out or uneven ion transport (see Figure S2). In contrast, GF membranes were developed for filtration applications and have an inhomogeneous surface and pore distribution, potentially contributing to uneven Zn deposition. The PP separators are designed to have a high mechanical strength and puncture resistance, which can suppress short circuits to some extent [39, 40, 41]. As shown in Figure S3 and Table S1, the PP separator exhibits substantially higher tensile strength and greater elongation at break than the GF separator, enabling it to better withstand the internal mechanical stresses generated during cycling. In addition, the PP separators are a mature industrial product that is cost‐efficient and manufactured in high yield with good batch consistency [42, 43]. However, as shown in Figure S4, the contact angle (CA) of aqueous electrolyte on the PP separator is 98°, demonstrating its typical hydrophobic nature. In contrast, typical carbonate (DMC) and ether (DME) based solvents exhibit low contact angles of ∼27°, indicating good wettability.

Organic/aqueous hybrid electrolytes were prepared by mixing deionized water with DMC or DME at organic solvent molar ratios of 20%, 25%, 33%, and 50%. The resulting water/DMC electrolytes were denoted as W20C, W25C, W33C, and W50C, while the corresponding water/DME electrolytes were denoted as W20E, W25E, W33E, and W50E, respectively. For comparison, 2 m Zn(OTf)_2_ electrolytes were fabricated using pure DMC, pure DME, and pure deionized water as solvents, denoted as 2mC, 2mE, and 2 mW, respectively. Digital photos of the obtained electrolytes show that 2mC and 2mE exhibit severe phase separation, indicating the poor solubility of Zn(OTf)_2_ in these solvents (Figure S5). After blending with water to increase the salt solubility, all the obtained electrolytes, except W50C, are homogeneous and transparent (Figure S6). We screened the wettability of the blended electrolytes on the PP separator using filter paper penetration tests. As shown in Figure S7, the obtained electrolytes were dropped onto a PP separator layer to evaluate their wettability. All blended electrolytes exhibited excellent wettability, readily penetrating the PP separator and completely wetting the underlying filter paper. To quantitatively evaluate electrolyte wettability, contact angle measurements were performed, as shown in Figure 1c and Figure S8. The CA values decrease progressively with increasing DMC/DME content. All hybrid electrolytes exhibit CAs below 70°, indicating markedly improved wettability toward the PP separator. The electrolyte uptake of the PP separator is closely correlated with its wettability in different electrolytes (see Table S2). As the organic‐solvent content increases, the wettability and electrolyte uptake of the PP separator increase substantially. Ignition tests confirmed that all electrolytes in the W50E–W25E series are flammable, whereas the W33C, W25C, and W20C electrolytes exhibit substantially reduced flammability and improved safety characteristics (Figures S9 and S10). The results indicate that DMC‐based electrolytes offer better safety performance than DME‐based systems while remaining compatible with commercially available PP separators and scalable large‐scale manufacturing.

To elucidate the relationship between solvent composition and electrolyte properties, electrolytes with varying DMC or DME contents were systematically characterized in terms of room‐temperature ionic conductivity, dynamic viscosity, and electrochemical stability window, as summarized in Tables S3 and S4 and Figure S11. These results demonstrate that increasing the proportion of DMC or DME gradually decreases the ionic conductivity and increases the viscosity of the electrolyte. The increased viscosity hinders ion transport, potentially leading to aggravated polarization during electrochemical cycling. On the other hand, the introduction of DMC/DME effectively extends the electrochemical stability window compared with the aqueous 2 mW electrolyte, indicating suppressed gas emission, which will be discussed in more detail further on.

Fourier transform infrared (FTIR) spectroscopy was performed to understand the water activity of the blended electrolytes. As shown in Figure 1d,e, in both blended electrolyte series, a blue shift of the O─H peak (3000–3600 cm^−1^) was observed upon the addition of DMC/DME solvents, indicating that the hydrogen‐bond network is weakened with the increasing amount of introduced organic solvent. In detail, the broadened O─H stretching vibration peak can be divided into three types of water species by Gaussian fitting, namely network water (NW) (∼3205 cm^−1^), intermediate water (IW) (∼3410 cm^−1^), and multimeric water (MW) (∼3560 cm^−1^) (see Figure S12) [44, 45]. Figure 1f summarizes the relative proportions of these three water species in the different electrolytes. The introduction of DMC/DME markedly disrupts the hydrogen‐bonding network, as evidenced by the pronounced reduction in the NW fraction from 46.3% in the aqueous electrolyte to 17.1% in both W50C and W50E. This disrupted hydrogen‐bond network in the blended electrolytes suggests suppressed anode degradation and parasitic HER, thereby enabling improved compatibility with Zn metal anodes. We took two pieces of zinc foil and soaked them separately in an aqueous electrolyte and a W33E electrolyte containing a moderate amount of organic solvent for 24 h. SEM images show that the Zn foil soaked in an aqueous electrolyte is covered with ZHS flakes, indicating severe Zn corrosion (Figure 1g). In contrast, Zn foil immersed in the W33C electrolyte exhibits markedly suppressed corrosion and retains a relatively flat and dense surface morphology (Figure 1h).

The Raman spectra of the H_2_O‐DMC electrolytes reveal the evolution of solvation structures (see Figure S13). Pure DMC exhibits a characteristic peak at approximately 916 cm^−1^, which is assigned to the C─O stretching vibration of the CH_3_─O group in uncoordinated DMC molecules [46, 47]. A new peak emerges at approximately 923 cm^−1^ in the H_2_O‐DMC hybrid electrolytes, which is attributed to DMC molecules coordinated with Zn^2+^. The relative fraction of Zn^2+^‐coordinated DMC gradually increases with increasing DMC content, as summarized in Table S5. For the H_2_O‐DME electrolytes, pure DME exhibits characteristic peaks at ∼821 and ∼849 cm^−1^ assigned to CH wagging coupled with C─O─C stretching [48]. Similarly, a new peak emerges at approximately 868 cm^−1^ in the hybrid electrolytes and is assigned to Zn^2+^‐coordinated DME. Its relative intensity increases with increasing DME content, indicating an enhanced contribution from DME‐ Zn^2+^ coordination.

Molecular dynamics (MD) simulations were conducted to elucidate the Zn^2^^+^ solvation structure in the optimized W33C hybrid electrolyte. Owing to the strong interactions among Zn^2+^, OTf^−^, and DMC, together with the reduced water content of the hybrid electrolyte, both DMC molecules and OTf^−^ anions participate in the primary solvation shell of Zn^2+^ (Figure S14). The reconstructed solvation environment is highly consistent with the spectral results obtained from FTIR and Raman tests (see Figures S12 and S13). Radial distribution functions (RDFs) were further analyzed to quantitatively elucidate the Zn^2+^ solvation configuration in the W33C electrolyte (Figure S15). The RDF results demonstrate that the primary solvation layer of Zn^2+^ is centered at approximately 0.2 nm. Obvious characteristic peaks assigned to Zn‐O (DMC) and Zn‐O (OTf^−^) bonds both appear at ∼0.2 nm, indicating the direct coordination of H_2_O, DMC, and OTf^−^ with Zn^2+^. Quantitative statistics show that the first solvation shell of Zn^2+^ in W33C electrolyte contains 3.39 H_2_O molecules, 0.66 DMC molecules, and 1.95 OTf^−^ anions on average, forming a hybrid solvation structure of Zn^2+^[H_2_O]_3.39_[DMC]_0.66_[OTf^−^]_1.95_. The co‐participation of OTf^−^ anions and DMC molecules in the solvation shell promotes unique interfacial chemistry through their reductive decomposition, likely facilitating the formation of a hybrid SEI layer on the Zn‐metal anode.

Zn//Zn symmetric cells were assembled to further evaluate the compatibility of the PP separator and blended electrolytes with Zn metal anodes (all symmetric cells were cycled at 1 mA·cm^−2^ and 3 mAh·cm^−2^). It is worth noting that the symmetric cells were assembled under relatively lean electrolyte conditions (electrolyte/test capacity = 10 g Ah^−1^), which would be insufficient to fully wet GFA membranes, but is sufficient for our thin PP separator. Figure 2a summarizes the cycling performance of Zn//Zn symmetric cells using the PP separator paired with the electrolytes discussed above. As expected, cells using the pure aqueous electrolyte cannot operate properly because of their poor PP wettability. Cells with blended electrolytes show significantly enhanced cycling stability. Specifically, both DME‐ and DMC‐blended electrolytes show a trend in which the cycle life first increases with the amount of added organic solvent and then decreases at elevated organic solvent content. W33C and W33E exhibit the best performance of their electrolyte formulations, achieving 4360 and 3337 h of operation before failure, respectively. As shown in Table S6, the cell employing the W33C electrolyte and PP separator outperforms most previously reported systems based on separator‐engineering strategies, particularly under practically relevant high‐areal‐capacity conditions. On the one hand, we know that increased solvent content leads to better separator wetting (see Figure 1c), but on the other hand, Figure 2b,c show that the cell polarization increases with organic solvent content in both DME‐ and DMC‐based electrolytes, indicating that the ionic conductivity decreases. Compared with W20E and W20C, the polarization of W50E and W50C increases by 88% and 237%, respectively. Therefore, it is expected that an intermediate solvent concentration leads to the best battery performance; this value could be further tweaked to meet certain cost or flammability targets.

**FIGURE 2:**
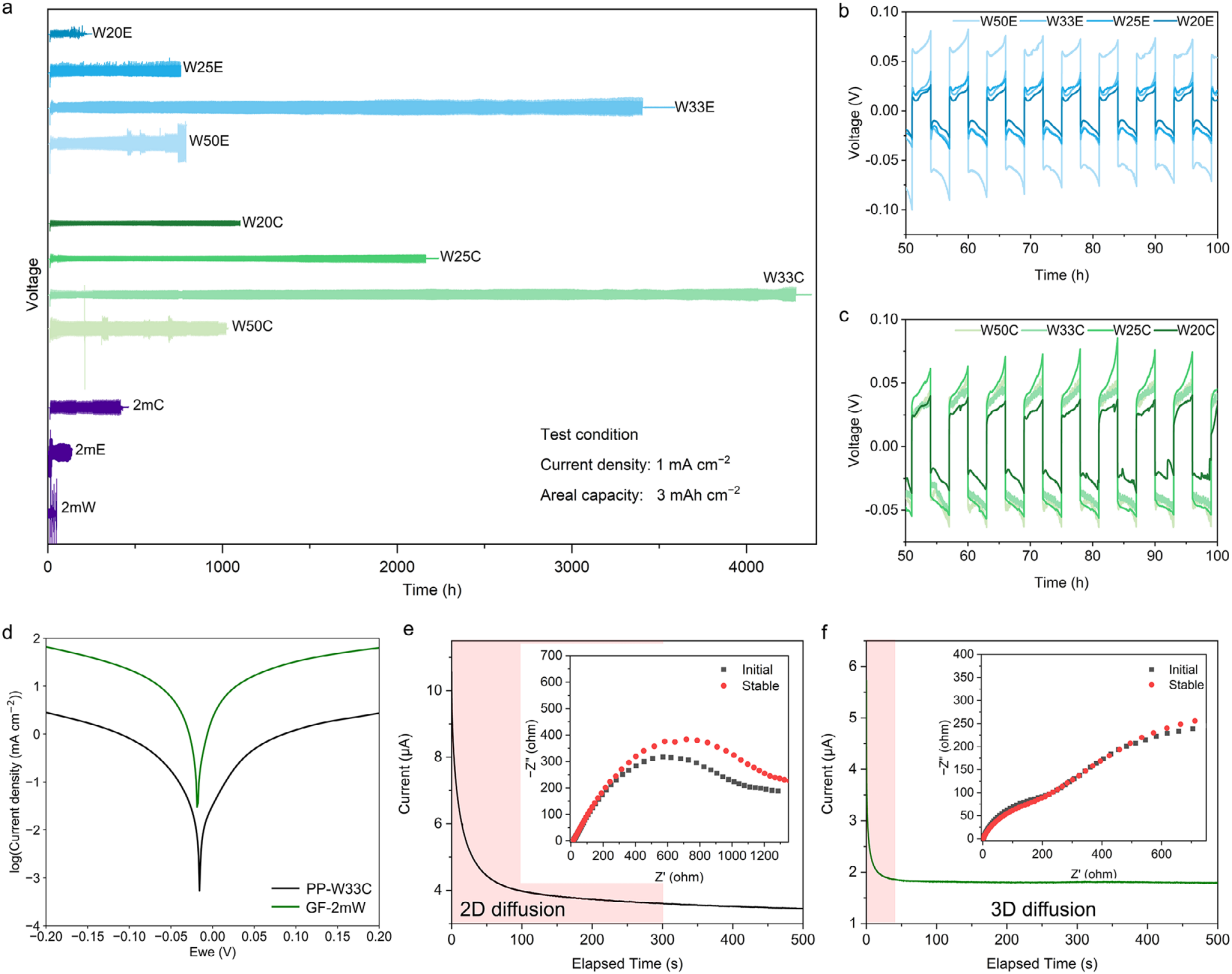
(a) Cycling performance of Zn//Zn symmetric cells assembled with the PP separator and different electrolytes. (b, c) Enlarged time–voltage profiles of Zn//Zn symmetric cells using (b) DME‐based electrolytes and (c) DMC‐based electrolytes. (d) Linear polarization curves of Zn//Zn symmetric cells using either the 2 mW electrolyte with the GF separator or the W33C electrolyte with the PP separator. (e, f) Electrochemical impedance spectroscopy (EIS) spectra collected before and after the chronoamperometry (CA) test for (e) a Zn//Zn symmetric cell with the GF separator and 2 mW electrolyte and (f) a Zn//Zn symmetric cell with the PP separator and W33C electrolyte.

In addition, Zn//Zn symmetric cells using the GF separator with an aqueous electrolyte and the W33C electrolyte were assembled for comparison. The cell using the W33C electrolyte and GF separator failed within 100 h, while the cell using the aqueous electrolyte and GF separator short‐circuited after 125 h (Figure S16). These results indicate that the marked improvement in cycling performance originates from the synergistic interplay between the hybrid organic‐aqueous electrolyte and the PP separator, rather than from either component alone.

Linear polarization measurements were conducted to assess the corrosion behavior of Zn metal anodes in the W33C electrolyte with the PP separator. As shown in Figure 2d, the cell assembled with the W33C electrolyte and PP separator exhibits a significantly reduced corrosion current and more positive corrosion potential than that of the counterpart using the aqueous electrolyte and GF separator, indicating suppressed Zn corrosion. An operando pressure test was conducted to verify the suppressed HER. As shown in Figure S17, the cell using the W33C electrolyte and PP separator exhibited only a slight pressure increase during the initial resting period, likely associated with SEI formation, with no further detectable pressure increase during subsequent cycling. After cycling, the PP separator maintained an intact morphology and uniform electrolyte distribution (Figure S18), whereas the GF separator in the aqueous electrolyte showed uneven electrolyte distribution and substantial by‐product accumulation.

Chronoamperometry (CA) and electrochemical impedance spectroscopy (EIS) tests were performed on Zn//Zn symmetric cells using different electrolytes and separators to investigate Zn plating/stripping behaviour. When a constant polarization voltage was applied, the cell using the aqueous electrolyte and the GF separator delivered a continuously decreasing current response over 300 s, indicating a prolonged two‐dimensional Zn^2+^ diffusion process (Figure 2e). By comparison, the cell employing the W33C electrolyte and PP separator reached a stable current response within 50 s, indicating a dominant three‐dimensional Zn^2+^ diffusion behaviour (Figure 2f). Moreover, the Zn^2+^ transference numbers ($t_{Zn^{2+}}$) for different electrolyte/separator configurations were determined from EIS measurements collected before and after the application of a constant polarization potential (see the inset plots in Figure 2e,f). The $t_{Zn^{2+}}$ for the W33C electrolyte with the PP separator ($t_{Zn^{2+}}=0.44$) is higher than that for the aqueous electrolyte with the GF separator ($t_{Zn^{2+}}=0.19$), further indicating optimized Zn^2+^ diffusion.

Post‐mortem SEM analyses were conducted to reveal the plating/stripping morphology of Zn metal anodes in different electrolytes and with different separators. As shown in Figure 3a–c, the Zn foils cycled in the W33C electrolyte with the PP separator exhibit dense and uniform plating morphologies over 3 mAh cm^−2^. In stark contrast, the Zn foil cycled in the aqueous electrolyte with the GF separator shows uneven Zn deposition as early as a deposited areal capacity of 1 mAh cm^−2^ (Figure 3d–f). After a 3 h deposition at 1 mA cm^−2^, Zn deposits propagate into the GF separator, which may eventually trigger a short circuit. Figure 3g shows that in similar cells cycled with W33C electrolyte with the PP separator, Zn deposits form a denser structure seemingly consisting of stacked hexagonal Zn plates. A cross‐sectional lamella of the deposited Zn was lifted out along the surface‐normal direction using focused ion beam (FIB) milling, as illustrated in Figure S19. The high‐angle annular dark‐field (HAADF) image of the deposited Zn grain shows a typical (002)‐oriented growth feature (see Figure 3h). In the literature, this preferential epitaxial growth of Zn along the (002) direction has been widely reported as an ideal crystallographic texture for stable cycling [49, 50], which is consistent with the markedly enhanced cyclability observed in Figure 2a. In addition, we observe substantial amounts of pits and cavities formed on the surface of the stripped Zn foil in the aqueous electrolyte with the GF separator (Figure 3i). This nonuniform surface condition is likely to trigger even more uneven Zn plating during the subsequent Zn plating process. In contrast, the stripped Zn disassembled from a cell using the W33C electrolyte and PP separator exhibits a relatively smooth morphology after stripping under the same test conditions (Figure 3j).

**FIGURE 3:**
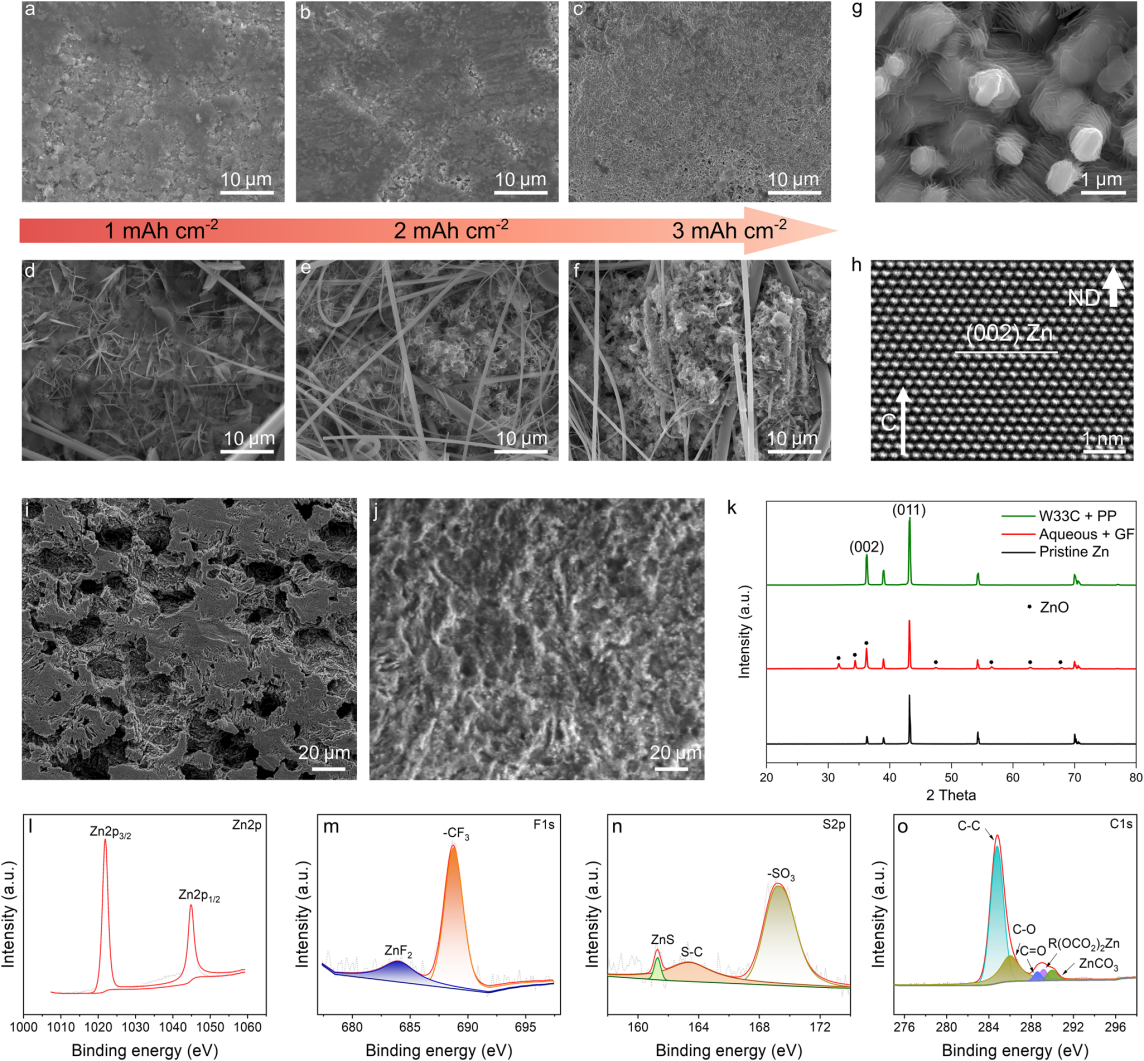
(a–f) SEM images of Zn metal electrodes after 1–3 h of Zn plating at 1 mA cm^−2^ in (a–c) the W33C electrolyte with the PP separator and (d–f) the aqueous electrolyte with the GF separator. (g) Enlarged SEM image of the plated Zn electrode. (h) High‐resolution HAADF image of the deposited Zn. (i, j) Post‐mortem SEM images of stripped Zn electrodes after 10 plating/stripping cycles at 1 mA cm^−2^ and 3 mAh cm^−2^ using (i) the aqueous electrolyte with the GF separator and (j) the W33C electrolyte with the PP separator. (k) XRD patterns of Zn electrodes after cycling in different electrolyte/separator configurations. (l–o) XPS spectra of the Zn metal anode after cycling in the W33C electrolyte with the PP separator.

XRD and XPS measurements were conducted to further understand the surface condition of the Zn metal anode cycled in the W33C electrolyte with the PP separator. As shown in Figure 3k, the Zn foil cycled for 10 cycles in the W33C electrolyte with the PP separator exhibits only characteristic Zn diffraction peaks, together with an increased (002)/(011) intensity ratio compared to the pristine Zn foil. It is worth noting that the (002) peak is not the dominant reflection in the XRD pattern, which is due to the random stacking and orientation distribution of the deposited Zn grains, even though local characterization reveals preferential growth along the (002) direction (see Figure 3g and Figure S20) [51]. In contrast, we observe intense additional peaks assigned to ZnO (01‐079‐0207) for the cycled Zn foil in the aqueous electrolyte with the GF separator. This is clear evidence that the Zn metal anode underwent passivation during electrochemical cycling in the aqueous electrolyte, whereas it remains predominantly metallic Zn in the W33C electrolyte. XPS results reveal the coexistence of inorganic species, including ZnF_2_ and ZnS, together with organic components on the surface of the Zn metal anodes cycled in W33C, indicating the formation of a hybrid organic–inorganic SEI layer (Figure 3l–o). Such a hybrid SEI layer has been widely recognized as beneficial for stabilizing Zn plating/stripping processes in previous studies [26, 52, 53].

Ar^+^ sputtering‐assisted XPS depth profiling further reveals the organic–inorganic gradient structure of the SEI formed on the cycled Zn anodes (Figure S21). The SEI comprises an outer layer rich in organic fragments derived from electrolyte decomposition and an inner inorganic layer consisting mainly of ZnCO_3_, ZnF_2_, and ZnS, with an overall thickness exceeding 100 nm. In many reported studies, the organic‐rich outer layer suppresses water permeation, whereas the inorganic‐rich inner layer, containing ZnS and ZnF_2_, facilitates rapid Zn^2^^+^ transport [54, 55, 56, 57].

A control experiment was conducted to verify the protective capability of the W33C‐derived SEI. Zn electrodes pre‐cycled in the W33C electrolyte to form the hybrid SEI were reassembled into cells containing the 2 mW electrolyte and GF separators for Tafel analysis, while Zn electrodes cycled directly in the 2 mW electrolyte served as the control. As shown in Figure S22, the SEI‐modified Zn electrode exhibited a significantly lower corrosion current density and a more positive corrosion potential, demonstrating that the hybrid interphase effectively isolates the Zn anode from the aqueous electrolyte and mitigates water‐induced parasitic reactions.

NaV_3_O_8_·1.5H_2_O (NVO) was synthesized as a cathode material to test the proposed electrolyte and separator in full cells. The crystal structure and morphology of the NVO cathode were systematically characterized via XRD, SEM, and EDS elemental mapping (Figures S23 and S24). These results verify the well‐defined crystalline phase, uniform microstructure and homogeneous elemental distribution of the as‐prepared NVO material. The NVO cathodes were first immersed in the aqueous and W33C electrolytes for 24 h. As shown in Figure 4a, the aqueous electrolyte turned yellow, and inductively coupled plasma (ICP) analysis shows that the dissolved V concentration in the W33C electrolyte is approximately one order of magnitude lower than that in the aqueous electrolyte after 24 h of soaking. These results indicate that the W33C electrolyte effectively suppresses vanadium dissolution, potentially leading to better cycling stability, in addition to the benefits of the anodes discussed above. As shown in Figure 4b, full cells using the W33C electrolyte and PP separator deliver a high specific capacity of 380 mAh g^−1^ and maintain relatively stable cycling performance over 150 cycles at 0.5 A g^−1^. In contrast, the counterpart using the aqueous electrolyte and GF separator suffers continuous capacity decay and fails within 120 cycles. Moreover, the full cell employing the W33C electrolyte and PP separator exhibits excellent high‐rate cycling performance, delivering a stable specific capacity of 225 mAh g^−1^ at 2 A g^−1^ over 1000 cycles (Figure 4c), corresponding to a capacity retention of 85% (based on 225 mAh g^−1^). In contrast, the counterpart using the aqueous electrolyte and GF separator retains only 22% of its capacity after 1000 cycles. Even at areal capacities of 4.4 mAh cm^−2^ (Figure 4d), the full cell employing the W33C electrolyte and PP separator achieves stable cycling, delivering 80% and 66% capacity retention over 200 cycles and 350 cycles, respectively. In stark contrast, the counterpart using the aqueous electrolyte and GF separator fails within only 32 cycles because of a soft short circuit (see the inset in Figure 4d).

**FIGURE 4:**
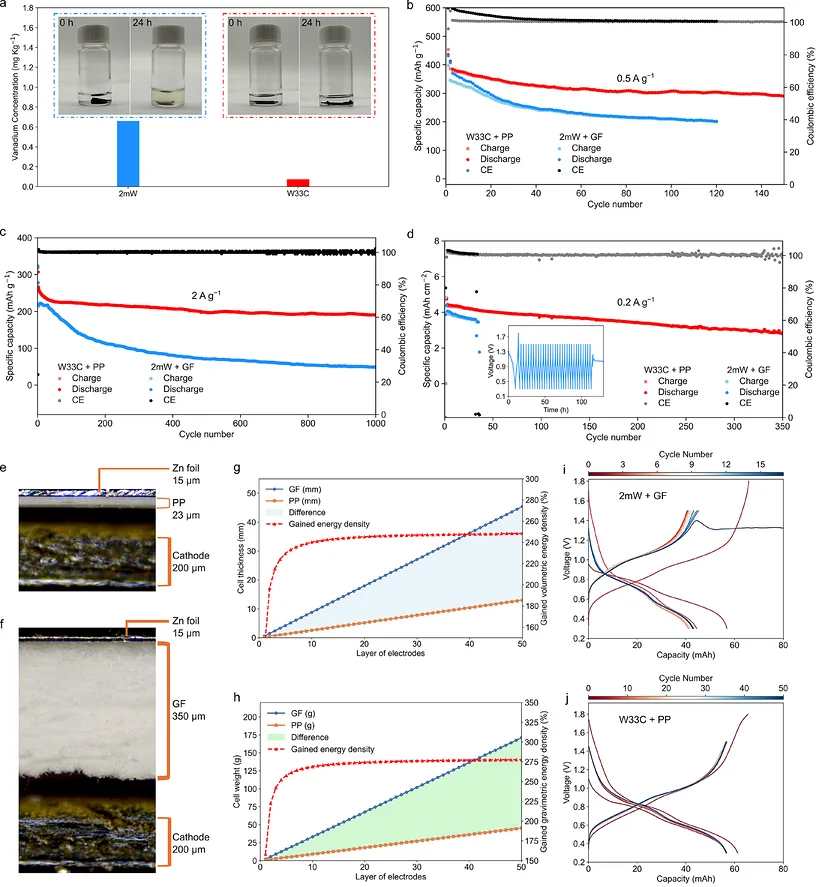
(a) Vanadium concentrations measured after 24 h of immersing NVO electrodes in different electrolytes. The inset shows optical photographs of the electrolytes before and after the 24 h soaking test. (b–d) Cycling performance of Zn//NVO full cells with different electrolyte/separator configurations at (b) 0.5 A g^−1^, (c) 2 A g^−1^, and (d) 0.2 A g^−1^ for high‐mass‐loading cathodes. The inset presents the time–voltage profile of a Zn//NVO full cell using the aqueous electrolyte and GF separator. (e, f) Cross‐sectional optical microscopy images of Zn//NVO full cells using (e) the PP separator and (f) the GF separator, together with the corresponding cell thicknesses. (g) Calculated thicknesses of Zn//NVO pouch cells with 1–50 electrode layers using different separators, along with the corresponding differences in volumetric energy density. (h) Calculated weights of Zn//NVO full cells with 1–50 electrode layers using different separators, together with the corresponding differences in gravimetric energy density. (i, j) Charge–discharge profiles of pouch cells employing (i) an aqueous electrolyte with a GF separator and (j) a W33C electrolyte with a PP separator.

Next, Zn//NVO pouch cells were assembled using a high‐massloading cathode (200 µm) and a thin Zn metal anode (15 µm) to analyse industrially viable cell configurations. Cross‐sectional optical microscopy images of pouch cells using different separators provide a direct comparison of the volume contributions of each component in a practical cell configuration (Figure 4e,f). The thin PP separator occupies only 9.7% of the total cell volume, whereas the GF separator accounts for a substantially larger volume fraction of 62%. In real‐world scenarios, 30–50 layers of electrode stacks are necessary to achieve a reasonable volumetric energy density. We calculated the cell thicknesses as well as the differences in volumetric energy density for cells using different separators based on Figure 4e,f for 1–50 layers of electrodes. As shown in Figure 4g and Table S7, the full cells using thin PP separators show a significantly decreased cell‐level thickness compared to those that use the GF separator when increasing the layers of electrodes. This calculation demonstrates that, when using the same anodes and cathodes, the PP separator could result in a 248% increase in volumetric energy density compared to the GF separator in a 50‐layer electrode configuration. In addition, the thin PP separators reduce the amount of electrolyte needed to wet the separator, which is critical for cell‐level gravimetric energy density. Similarly, we calculated the corresponding gains in gravimetric energy density under the same conditions upon replacing the GF separator with the PP separator. This results in a 278% improvement, as shown in Figure 4h and Table S8. Importantly, these gains in energy density simultaneously allow improving the lifetime of pouch cells by threefold (Figure 4i,j), which demonstrates that combined electrolyte and separator optimisation is a promising approach to improve both the energy density and lifetime of ZIBs.

## 3 Conclusions

In summary, thin separators that enable well‐regulated Zn plating/stripping behavior are essential for achieving high volumetric and gravimetric energy densities in ZIBs. The widely used GF separators are not only too bulky to enable satisfactory energy densities, but also lead to nonuniform Zn plating. Here we show how hydrophobic PP separators developed for LIBs can be leveraged to make better ZIBs by using hybrid organic/aqueous electrolytes. Through the rational design of the latter, anode and cathode degradation can be simultaneously suppressed, while the wettability of the thin PP separators is improved, thereby promoting uniform Zn plating/stripping. As a result, the Zn//Zn symmetric cell employing the W33C electrolyte and PP separator delivers an outstanding cycling lifetime of 4360 h at a practical areal capacity of 3 mAh cm^−2^. Further, the Zn//NVO full cell delivers stable cycling performance over 200 cycles at a high areal capacity of 4.4 mAh cm^−2^, which is almost an order of magnitude higher than reference conditions. Along with a two‐fold increase in energy density, these cells demonstrate that a synergistic design of electrolytes and separators is an overlooked strategy to design next generation ZIBs.

## Author Contributions

**Gao Wang**: investigation, writing – original draft, methodology, validation, visualization, data curation, formal analysis. **Ze He**: conceptualization, methodology, data curation, investigation, validation, formal analysis, supervision, visualization, writing – original draft, writing – review and editing. **Rui Wang**: investigation, validation, formal analysis. **Jiajing Wang**: investigation, validation, formal analysis. **Shaowen Cao**: formal analysis, supervision, writing – review and editing. **Michael De Volder**: validation, formal analysis, funding acquisition, writing – review and editing, resources. **Qinyou An**: validation, investigation, supervision, resources, funding acquisition, writing – review and editing.

## Conflicts of Interest

The authors declare no conflicts of interest.

## Supporting information




**Supporting File**: advs78250‐sup‐0001‐SuppMat.docx

## Data Availability

The data that support the findings of this study are available in the supplementary material of this article.
