# Supplementary material for: Toward Practical Zn‐Ion Batteries Through Separator/Electrolyte Co‐Design
Source: Adv Sci (Weinh). 2026 Oct 29:e78250. Online ahead of print. doi: 10.1002/advs.78250 (PMC13720260; doi:10.1002/advs.78250)
Supplement: Supplementary file 1 [file ADVS-9999-e78250-s001.docx]

Supplementary Information

**Toward Practical Zn-Ion Batteries through Separator/Electrolyte Co-Design**

Gao Wang^1^, Ze He^2^*, Rui Wang^2^, Jiajing Wang^1^, Shaowen Cao^1^, Michael De Volder^2^*, Qinyou An^1^*

^1^Key Laboratory of Advanced Technology for Materials Synthesis and Processing, Wuhan University of Technology, Wuhan, 430070, China

^2^Institute for Manufacturing, Department of Engineering, University of Cambridge, Cambridge CB3 0FS, UK

*Corresponding authors (email: zh360@cam.ac.uk; [anqinyou86@whut.edu.cn](mailto:anqinyou86@whut.edu.cn); [mfld2@cam.ac.uk](mailto:mfld2@cam.ac.uk))

**Experimental Section**

**Materials**

The Zn(OTf)_2_-based aqueous electrolytes with different amount of DMC/DME additives were prepared by dissolving the desired amount of salts into the selected solvents. The molar fractions of organic solvents (DMC/DME) are controlled at 20%, 25%, 33%, and 50%, corresponding to water/organic solvent molar ratios of 4:1, 3:1, 2:1, and 1:1, respectively. Accordingly, the as-prepared electrolytes are designated as W20C/W20E, W25C/W25E, W33C/W33E, and W50C/W50E. The aqueous 2 m Zn(OTf)_2_ aqueous electrolyte without adding DMC/DME is denoted as 2mW. Taking the W33C electrolyte as an example, H_2_O and DMC solvents with a molar ratio of 2:1 were mixed firstly, and then 2 m Zn(OTf)_2_ was dissolved into the bi-solvent system. Synthesis of NaV_3_O_8_ •1.5 H_2_O (NVO) nanobelts: 4 grams of V_2_O_5_ powder and 120 mL carbon nanotube dispersion (0.4 wt% CNT and 0.6 wt% CMC) was added into 120 mL of 2 M NaCl aqueous solution. After stirring for 96 h at 30 ℃, the suspension was washed with deionized water for several times. Finally, the black red product was obtained by freeze-drying for 3 days. To prepare the freestanding electrode, 4 g of V_2_O_5_ powder and 120 mL of a carbon nanotube dispersion (0.4 wt% CNT and 0.6 wt% CMC) were added to 120 mL of 2 M NaCl aqueous solution and stirred for 4 days. The resulting slurry was then vacuum-filtered onto filter paper to form a film (~200 μm), followed by freeze-drying for 48 h to obtain the freestanding electrode.

**Materials Characterization**

The samples were characterized by an X-ray diffractometer (BrukerD2 Phaser, Cu Kα radiation). The morphology of the samples was observed using a field-emission scanning electron microscope (SEM, JEOL JSM-7100F). Transmission electron microscope (TEM) images, high angle annular dark field imaging-scanning TEM (HAADF-STEM) images of the samples were characterized by a double spherical aberration corrected transmission electron microscope (Titan Themis G2 60-300, Thermo Fisher). Focused ion beam (FIB) characterization of the samples was carried out on a focused ion beam scanning electron microscope (Helios NanoLab G3 UC, Thermo Fisher Scientific). Fourier transform infrared (FT-IR) transmittance spectra and Raman spectra were acquired using a Nicolet 6700 (Thermo Fisher Scientific Co., USA) IR spectrometer and HORIBA HR EVO Raman system with an excitation laser of 633 nm, respectively. Ex situ X-ray photoelectron spectroscopy (XPS) spectra were recorded using a Kratos AXIS SUPRA spectrometer. Contact angle tests were measured on Dataphysics OCA35 optical contact angle system.

**Electrochemical tests**

Symmetric Zn/Zn cells were employed to study the cycling performance of H_2_O+DMC/DME electrolytes. LSV measurements were carried out in a Zn||Zn symmetric cell configuration. Electrochemical performances of Zn-ion full cells were evaluated using CR2025 coin-type cells with NVO cathode, Polypropylene (Celgard2500) separator, and Zn foil anode. For coin cells, the working electrode was fabricated by blending active materials powder, acetylene black, Polytetrafluoroethylene (PTFE) in a weight ratio of 6:3.5:0.5. The Zn electrode was cut into round slices with a diameter of 10 mm and a thickness of 53 μm. For pouch-cell assembly, the as-prepared freestanding NVO electrode with a mass loading of 12.5 mg cm^−2^ was used as the cathode, while 15 μm Zn foil was used as the anode. EIS was performed on a CHI660S-3 electrochemical work station. Charge/discharge of battery was tested on an LAND-CT2001A battery-testing instrument. Calculation of specific capacity and current density was based on the active cathode mass. The Zn^2+^ transference number of different separators was analyzed by conducting EIS measurements before and after a constant polarization potential of 15 mV. Based on the following calculation:

$$t^{+}=\frac{I_{s}(\Delta V- I_{0}R_{0})}{I_{0}(\Delta V- I_{s}R_{s})}$$

Where *I_0_* and *I_s_* represent the initial and steady-state currents, respectively, *ΔV* refers to the applied constant potential of 15 mV, and *R_0_* and *R_s_* correspond to the initial and steady-state charge-transfer resistances, respectively.

**Figure S1.** Thickness Measurement of GFA Separator(a) and PP Separator(b).

**Figure S2.** Scanning electron microscopy (SEM) images of PP and GFA separators.

(a, b) Low- and high-magnification SEM images of the PP separator, showing its dense, elongated pore structure (scale bars: 10 μm and 1 μm, respectively).

(c, d) Low- and high-magnification SEM images of the GFA separator (scale bars: 10 μm and 1 μm, respectively).

**Figure S3.** Tensile stress–strain curves of commercial PP and GFA separators.

**Figure S4.** Static contact angles of different electrolytes on PP separator.

(a) 2mW electrolyte; (b) 2mC electrolyte; (c) 2mE electrolyte.

**Figure S5.** Optical photos of 2mW, 2mC, and 2mE electrolytes.

**Figure S6.** Optical photos of (a) W50C-W20C electrolytes and (b) W50E-W20E electrolytes.

**Figure S7.** Wettability test of different electrolytes on the PP separator.

(a) Droplet behavior of the 2mW electrolyte on the separator and the corresponding wetting state of the underlying filter paper. (b) Wettability of the W50C–W20C electrolyte series. (c) Wettability of the W50E–W20E electrolyte series. The left panels show the droplet morphology on the separator surface, while the right panels show the corresponding wetting footprints on the filter paper beneath.

**Figure S8.** Contact angle measurements of (a) W50C/W33C/W25C/W20C and (b) W50E/W33E/W25E/W20E electrolytes.

**Figure S9.** Flammability tests of W50E-W20E electrolytes (W50E, W33E, W25E, and W20E from top to bottom)**.**

**Figure S10.** Flammability tests of W50C-W20C electrolytes (W50C, W33C, W25C, and W20C from top to bottom).

**Figure S11.** Electrochemical stability windows measured by LSV for different hybrid electrolytes. (a) Series of DMC-containing electrolytes (W20C, W25C, W33C, W50C) and 2 mW electrolyte; (b) Series of DME-containing electrolytes (W20E, W25E, W33E, W50E) and 2 mW electrolyte.

**Figure S12.** FTIR spectra and corresponding fitted curves of different electrolytes: (a) W20C, W25C, W33C and W50C (b) W20E, W25E, W33E and W50E.

**Figure S13.** Raman spectra for (a) H_2_O-DMC electrolytes and (b) H_2_O-DME electrolytes.

**Figure S14.** Snapshot of the MD simulation box and representative Zn^2+^ solvation structure for the W33C electrolyte.

**Figure S15.** RDFs and coordination numbers of Zn^2+^ paired with H_2_O, DMC and OTf^−^ in the W33C electrolyte.

**Figure S16.** Galvanostatic polarization curve of the Zn||Zn symmetric cell with 2mW/W33C electrolytes at 1 mA cm^−2^, 3mA h cm^−2^.

**Figure S17.** Pressure-time and voltage-time curves of Zn symmetrical cells using the W33C electrolyte and the PP separator.

**Figure S18.** Digital photos of (a) the cycled PP separator with the W33C electrolyte and (b) the cycled GFA separator with aqueous electrolyte.

**Figure S19.** FIB-SEM images of (a) the top-view and (b) the cross-sectional lamella (lifted out by FIB milling) of the deposited Zn.

**Figure S20.** Ex-situ SEM image of the Zn deposition surface after 10 cycles at 1 mA cm^-2^, 3 mA h cm^-2^**.**

 **Figure S21.** Ar⁺ sputtering XPS depth profiling spectra (C 1s, F 1s, S 2p, Zn 2p) of the Zn anode after 10 cycles in the W33C electrolyte.

**Figure S22.** Tafel polarization curves of Zn electrodes pre-cycled for three cycles in either the W33C electrolyte with a PP separator or the 2mW electrolyte with a GF separator (test condition: 1 mA cm^−2^, 3 mAh cm^−2^). After pre-cycling, both Zn electrodes were tested in the 2mW electrolyte using GF separators under identical conditions.

**Figure S23.** (a) XRD pattern and (b) SEM image of the synthesized NVO@CNT cathode material. Scale bar, 0.5 μm.

**Figure S24.** SEM and EDS elemental mapping (Na, V, O) of the NVO@CNT cathode. Scale bar, 10 μm.

**Table S1.** Comparison of PP and GFA separators.

| Parameter | PP | GFA |
| --- | --- | --- |
| Thickness | 23 μm | 350 μm |
| Average pore size | ~0.064 μm | ~1.5 μm |
| Porosity | 40-45% | 75-90% |
| Tensile strength | 30.64 MPa | 0.96 MPa |
| Young’s modulus | 415.27 MPa | 144.81 MPa |

**Table S2.** Electrolyte uptake of PP separator towards various electrolytes.

| Electrolyte | Mass before immersion | Mass after immersion | Electrolyte uptake |
| --- | --- | --- | --- |
| W50C | 7.10 mg | 124.01 mg | 1646% |
| W33C | 7.12 mg | 36.05 mg | 406% |
| W25C | 7.06 mg | 23.03 mg | 226% |
| W20C | 7.15 mg | 27.17 mg | 280% |
| W50E | 7.15 mg | 65.01 mg | 809% |
| W33E | 7.17 mg | 50.91 mg | 610% |
| W25E | 7.25 mg | 47.12 mg | 549% |
| W20E | 7.16 mg | 37.15 mg | 418% |

**Table S3.** The ionic conductivity values of the hybrid electrolytes.

| Electrolyte | Test1 | Test2 | Test3 | Test4 | Test5 | Average |
| --- | --- | --- | --- | --- | --- | --- |
| W50E | 10.60 | 10.50 | 10.47 | 10.44 | 10.43 | 10.49 |
| W33E | 14.05 | 14.01 | 13.97 | 13.94 | 13.93 | 13.98 |
| W25E | 17.47 | 17.38 | 17.32 | 17.29 | 17.26 | 17.34 |
| W20E | 20.87 | 20.78 | 20.74 | 20.69 | 20.65 | 20.75 |
| W50C | 6.39 | 6.36 | 6.28 | 6.29 | 6.27 | 6.32 |
| W33C | 14.37 | 14.27 | 14.24 | 14.23 | 14.22 | 14.27 |
| W25C | 24.86 | 24.76 | 24.70 | 24.67 | 24.62 | 24.72 |
| W20C | 26.78 | 26.67 | 26.62 | 26.57 | 26.55 | 26.64 |
| 2mW | 41.20 | 40.50 | 40.29 | 40.07 | 40.18 | 40.45 |

Note: The unit of ionic conductivity is mS cm^−1^

**Table S4.** Dynamic viscosity of various electrolytes (25 ℃, unit: mPa ∙ s)

| Electrolyte | Test1 | Test2 | Test3 | Average |
| --- | --- | --- | --- | --- |
| W50E | 1.33 | 1.35 | 1.36 | 1.35 |
| W33E | 1.25 | 1.24 | 1.25 | 1.25 |
| W25E | 1.19 | 1.17 | 1.16 | 1.17 |
| W20E | 1.17 | 1.15 | 1.13 | 1.15 |
| W50C | 1.42 | 1.44 | 1.42 | 1.43 |
| W33C | 1.33 | 1.35 | 1.33 | 1.34 |
| W25C | 1.27 | 1.28 | 1.29 | 1.28 |
| W20C | 1.25 | 1.23 | 1.24 | 1.24 |
| 2mW | 1.91 | 1.93 | 1.88 | 1.91 |

**Table S5.** Peak-fitting results of the Raman spectra for the H_2_O–DMC electrolytes.

| Electrolyte | Free DMC | DMC-Zn^2+^ |
| --- | --- | --- |
| DMC | 100% | 0 % |
| W50C | 21.4% | 78.6% |
| W33C | 35.9% | 64.1% |
| W25C | 38.5% | 61.5% |
| W20C | 40.1% | 59.9% |

**Table S6.** Comparison of symmetric cell performance of reported separators for aqueous zinc metal batteries

| Separator | Modification | Method | Symmetrical cell performance |
| --- | --- | --- | --- |
| PP | / | Hybrid electrolyte | 4360 h at 1 mA cm^-2^,  3 mA h cm^-2^ |
| Cellulose | Zr-CNF^[1]^ | In-situ hydrolysis | 700 h at 10 mA cm^-2^,  2 mA h cm^-2^ |
| Cellulose | Janus(AgNWs/BC)^[2]^ | Filtration | Over 1000h at 80 mA cm^-2^,  1 mA h cm^-2^ |
| Cellulose | CNF+LMS^[3]^ | Solution casting | 1000 h at 1 mA cm^-2^,  0.5 mA h cm^-2^ |
| Cellulose | FACNF^[4]^ | Dip–coating | 1600 h at 2 mA cm^-2^,  1 mA h cm^-2^ |
| Cellulose | ZC^[5]^ | Solution casting | 2000 h at 0.5 mA cm^-2^,  0.25 mA h cm^-2^ |
| GF | Mxene@NiO@GF^[6]^ | Slurry coating | Over 2400 h at 2 mA cm^-2^,  2 mA h cm^-2^ |
| GF | ZnHAP/BC^[7]^ | nature-inspired | Over 1600 h at 1 mA cm^−2^,  1 mA h cm^-2^ |
| GF | NPGO@GF^[8]^ | Spray coating | Over 300 h at 20 mA cm^-2^,  1 mA h cm^-2^ |
| GF | Janus(SPAES@GF)^[9]^ | Coating | Over 2000 h at 1 mA cm^-2^,  1 mA h cm^-2^ |
| GF | PVDF@GF^[10]^ | Grafting | 500 h at 10 mA cm^-2^,  1 mA h cm^-2^ |

**Table S7.** Differences in separator thickness, resulting cell volume, and energy density for 1–50 layers pouch cells using different separators are compared.

Specifically, the thicknesses of the cathode (including current collector), anode, GF separator, and PP separator are 200, 15, 350, and 23 μm, respectively. The N/P ratio is 2, and the cathode mass loading is 12.5 mg cm^-2^.

| Layer of electrodes | GF cell(mm) | PP cell(mm) | Difference (mm) | Volume reduce | Energy density increase |
| --- | --- | --- | --- | --- | --- |
| 1 | 0.565 | 0.238 | 0.327 | 57.88% | 137.39% |
| 2 | 1.48 | 0.499 | 0.981 | 66.28% | 196.59% |
| 3 | 2.395 | 0.76 | 1.635 | 68.27% | 215.13% |
| 4 | 3.31 | 1.021 | 2.289 | 69.15% | 224.19% |
| 5 | 4.225 | 1.282 | 2.943 | 69.66% | 229.56% |
| 6 | 5.14 | 1.543 | 3.597 | 69.98% | 233.12% |
| 7 | 6.055 | 1.804 | 4.251 | 70.21% | 235.64% |
| 8 | 6.97 | 2.065 | 4.905 | 70.37% | 237.53% |
| 9 | 7.885 | 2.326 | 5.559 | 70.50% | 238.99% |
| 10 | 8.8 | 2.587 | 6.213 | 70.60% | 240.16% |
| 11 | 9.715 | 2.848 | 6.867 | 70.69% | 241.11% |
| 12 | 10.63 | 3.109 | 7.521 | 70.75% | 241.91% |
| 13 | 11.545 | 3.37 | 8.175 | 70.81% | 242.58% |
| 14 | 12.46 | 3.631 | 8.829 | 70.86% | 243.16% |
| 15 | 13.375 | 3.892 | 9.483 | 70.90% | 243.65% |
| 16 | 14.29 | 4.153 | 10.137 | 70.94% | 244.09% |
| 17 | 15.205 | 4.414 | 10.791 | 70.97% | 244.47% |
| 18 | 16.12 | 4.675 | 11.445 | 71.00% | 244.81% |
| 19 | 17.035 | 4.936 | 12.099 | 71.02% | 245.12% |
| 20 | 17.95 | 5.197 | 12.753 | 71.05% | 245.38% |
| 21 | 18.865 | 5.458 | 13.407 | 71.07% | 245.63% |
| 22 | 19.78 | 5.719 | 14.061 | 71.09% | 245.85% |
| 23 | 20.695 | 5.98 | 14.715 | 71.10% | 246.04% |
| 24 | 21.61 | 6.241 | 15.369 | 71.12% | 246.23% |
| 25 | 22.525 | 6.502 | 16.023 | 71.13% | 246.39% |
| 26 | 23.44 | 6.763 | 16.677 | 71.15% | 246.54% |
| 27 | 24.355 | 7.024 | 17.331 | 71.16% | 246.68% |
| 28 | 25.27 | 7.285 | 17.985 | 71.17% | 246.80% |
| 29 | 26.185 | 7.546 | 18.639 | 71.18% | 246.92% |
| 30 | 27.1 | 7.807 | 19.293 | 71.19% | 247.03% |
| 31 | 28.015 | 8.068 | 19.947 | 71.20% | 247.13% |
| 32 | 28.93 | 8.329 | 20.601 | 71.21% | 247.22% |
| 33 | 29.845 | 8.59 | 21.255 | 71.22% | 247.30% |
| 34 | 30.76 | 8.851 | 21.909 | 71.23% | 247.38% |
| 35 | 31.675 | 9.112 | 22.563 | 71.24% | 247.46% |
| 36 | 32.59 | 9.373 | 23.217 | 71.24% | 247.53% |
| 37 | 33.505 | 9.634 | 23.871 | 71.25% | 247.59% |
| 38 | 34.42 | 9.895 | 24.525 | 71.25% | 247.65% |
| 39 | 35.335 | 10.156 | 25.179 | 71.26% | 247.71% |
| 40 | 36.25 | 10.417 | 25.833 | 71.26% | 247.77% |
| 41 | 37.165 | 10.678 | 26.487 | 71.27% | 247.82% |
| 42 | 38.08 | 10.939 | 27.141 | 71.27% | 247.87% |
| 43 | 38.995 | 11.2 | 27.795 | 71.28% | 247.91% |
| 44 | 39.91 | 11.461 | 28.449 | 71.28% | 247.96% |
| 45 | 40.825 | 11.722 | 29.103 | 71.29% | 248.00% |
| 46 | 41.74 | 11.983 | 29.757 | 71.29% | 248.33% |
| 47 | 42.655 | 12.244 | 30.411 | 71.30% | 248.37% |
| 48 | 43.57 | 12.505 | 31.065 | 71.30% | 248.42% |
| 49 | 44.485 | 12.766 | 31.719 | 71.30% | 248.46% |
| 50 | 45.4 | 13.027 | 32.373 | 71.31% | 248.51% |

**Table S8.** Comparison of separator mass, electrolyte uptake mass, resulting cell mass, and energy density for pouch cells with 1–50 electrode layers (cell area: 20 cm²) using different separators.

Specifically, the masses of the cathode (mass loading: 12.5 mg cm⁻²), 10 μm stainless steel current collector, 15 μm Zn anode, GF separator with absorbed electrolyte (after pressing), and PP separator with absorbed electrolyte (after pressing) are 315, 168, 214, 1374, and 105 mg, respectively.

| Layer of electrodes | GF cell (mg) | PP cell (mg) | Difference (mg) | Mass reduce | Energy density increase |
| --- | --- | --- | --- | --- | --- |
| 1 | 2071 | 802 | 1269 | 61.27% | 158.23% |
| 2 | 5516 | 1709 | 3807 | 69.02% | 222.76% |
| 3 | 8961 | 2616 | 6345 | 70.81% | 242.55% |
| 4 | 12406 | 3523 | 8883 | 71.60% | 252.14% |
| 5 | 15851 | 4430 | 11421 | 72.05% | 257.81% |
| 6 | 19296 | 5337 | 13959 | 72.34% | 261.55% |
| 7 | 22741 | 6244 | 16497 | 72.54% | 264.21% |
| 8 | 26186 | 7151 | 19035 | 72.69% | 266.19% |
| 9 | 29631 | 8058 | 21573 | 72.81% | 267.72% |
| 10 | 33076 | 8965 | 24111 | 72.90% | 268.95% |
| 11 | 36521 | 9872 | 26649 | 72.97% | 269.95% |
| 12 | 39966 | 10779 | 29187 | 73.03% | 270.78% |
| 13 | 43411 | 11686 | 31725 | 73.08% | 271.48% |
| 14 | 46856 | 12593 | 34263 | 73.12% | 272.08% |
| 15 | 50301 | 13500 | 36801 | 73.16% | 272.60% |
| 16 | 53746 | 14407 | 39339 | 73.19% | 273.05% |
| 17 | 57191 | 15314 | 41877 | 73.22% | 273.46% |
| 18 | 60636 | 16221 | 44415 | 73.25% | 273.81% |
| 19 | 64081 | 17128 | 46953 | 73.27% | 274.13% |
| 20 | 67526 | 18035 | 49491 | 73.29% | 274.42% |
| 21 | 70971 | 18942 | 52029 | 73.31% | 274.68% |
| 22 | 74416 | 19849 | 54567 | 73.33% | 274.91% |
| 23 | 77861 | 20756 | 57105 | 73.34% | 275.13% |
| 24 | 81306 | 21663 | 59643 | 73.36% | 275.32% |
| 25 | 84751 | 22570 | 62181 | 73.37% | 275.50% |
| 26 | 88196 | 23477 | 64719 | 73.38% | 275.67% |
| 27 | 91641 | 24384 | 67257 | 73.39% | 275.82% |
| 28 | 95086 | 25291 | 69795 | 73.40% | 275.97% |
| 29 | 98531 | 26198 | 72333 | 73.41% | 276.10% |
| 30 | 101976 | 27105 | 74871 | 73.42% | 276.23% |
| 31 | 105421 | 28012 | 77409 | 73.43% | 276.35% |
| 32 | 108866 | 28919 | 79947 | 73.43% | 276.46% |
| 33 | 112311 | 29826 | 82485 | 73.44% | 276.57% |
| 34 | 115756 | 30733 | 85023 | 73.45% | 276.67% |
| 35 | 119201 | 31640 | 87561 | 73.46% | 276.76% |
| 36 | 122646 | 32547 | 90099 | 73.46% | 276.85% |
| 37 | 126091 | 33454 | 92637 | 73.47% | 276.94% |
| 38 | 129536 | 34361 | 95175 | 73.47% | 277.02% |
| 39 | 132981 | 35268 | 97713 | 73.48% | 277.10% |
| 40 | 136426 | 36175 | 100251 | 73.48% | 277.17% |
| 41 | 139871 | 37082 | 102789 | 73.49% | 277.24% |
| 42 | 143316 | 37989 | 105327 | 73.49% | 277.31% |
| 43 | 146761 | 38896 | 107865 | 73.50% | 277.37% |
| 44 | 150206 | 39803 | 110403 | 73.50% | 277.43% |
| 45 | 153651 | 40710 | 112941 | 73.50% | 277.49% |
| 46 | 157096 | 41617 | 115479 | 73.51% | 277.55% |
| 47 | 160541 | 42524 | 118017 | 73.51% | 277.60% |
| 48 | 163986 | 43431 | 120555 | 73.51% | 277.65% |
| 49 | 167431 | 44338 | 123093 | 73.52% | 277.70% |
| 50 | 170876 | 45245 | 125631 | 73.52% | 277.75% |

**Table S9.** Calculation of negative-to-positive (N/P) capacity ratio.

| Zn Thickness | Zn theoretical specific capacity | Zn anode areal capacity |
| --- | --- | --- |
| 15 μm | 820 mA h g^-1^ | 8.782 mA h cm^-2^ |
| Cathode specific capacity | Cathode active areal loading | Cathode areal capacity |
| 350 mA h g^-1^ | 12.5 mg cm^-2^ | 4.375 mA h cm^-2^ |

The N/P ratio (negative/positive capacity ratio) is defined as the capacity ratio of the negative electrode to the positive electrode. The calculation formula is shown below:

$$\frac{N}{P}=\frac{Areal capacity\left( anode \right)}{Areal capacity\left( cathode \right)}=\frac{8.782m Ah cm⁻^{2}}{4.375m Ah cm⁻^{2}}=2.01$$

Regarding the electrolyte amount, we fixed the electrolyte-to-capacity (E/C) ratio at 10 g Ah^−1^. For full cells, the electrolyte amount was determined based on the cathode capacity, whereas for symmetric cells, it was calculated based on the capacity tested per cycle (3 mAh cm^−2^ × 0.785 cm^2^).

1. S. Yang, Y. Zhang, Y. Zhang, et al., “Designing Anti‐Swelling Nanocellulose Separators with Stable and Fast Ion Transport Channels for Efficient Aqueous Zinc‐Ion Batteries,” *Advanced Functional Materials* *33*, no. 42 (2023): 2304280, https://doi.org/10.1002/adfm.202304280.

2. Z. Zheng, S. Guo, M. Yan, Y. Luo, and F. Cao, “A Functional Janus Ag Nanowires/Bacterial Cellulose Separator for High‐Performance Dendrite‐Free Zinc Anode Under Harsh Conditions,” *Advanced Materials* *35*, no. 47 (2023): 2304667, https://doi.org/10.1002/adma.202304667.

3. J. Cao, D. Zhang, R. Chanajaree, et al., “A Low-Cost Separator Enables a Highly Stable Zinc Anode by Accelerating the de-Solvation Effect,” *Chemical Engineering Journal* *480* (2024): 147980, https://doi.org/10.1016/j.cej.2023.147980.

4. C. Yang, P. Woottapanit, Y. Yue, et al., “Industrial Waste Derived Separators for Zn‐Ion Batteries Achieve Homogeneous Zn(002) Deposition Through Low Chemical Affinity Effects,” *Small* *20*, no. 26 (2024): 2311203, https://doi.org/10.1002/smll.202311203.

5. J. Cao, D. Zhang, C. Gu, et al., “Modulating Zn Deposition via Ceramic-Cellulose Separator with Interfacial Polarization Effect for Durable Zinc Anode,” *Nano Energy* *89* (2021): 106322, https://doi.org/10.1016/j.nanoen.2021.106322.

6. Y. An, Y. Tian, Q. Man, et al., “Highly Reversible Zn Metal Anodes Enabled by Freestanding, Lightweight, and Zincophilic MXene/Nanoporous Oxide Heterostructure Engineered Separator for Flexible Zn-MnO2 Batteries,” *ACS Nano* *16*, no. 4 (2022): 6755–6770, https://doi.org/10.1021/acsnano.2c01571.

7. H. Qin, W. Chen, W. Kuang, et al., “A Nature-Inspired Separator with Water-Confined and Kinetics-Boosted Effects for Sustainable and High-Utilization Zn Metal Batteries,” *Small* *19*, no. 20 (2023): 2300130, https://doi.org/10.1002/smll.202300130.

8. K. Zhu, L. Wu, C. Guo, et al., “Multiscale Ion‐Sieving Separator with Selective Zn^2+^ Channels and Excellent Zn^2+^ Desolvation Kinetics for Dendrite‐Free and Kinetics‐Enhanced Zinc Metal Batteries,” *Advanced Functional Materials* *33*, no. 47 (2023): 2305098, https://doi.org/10.1002/adfm.202305098.

9. Y. Sun, Q. Jian, T. Wang, et al., “A Janus Separator towards Dendrite-Free and Stable Zinc Anodes for Long-Duration Aqueous Zinc Ion Batteries,” *Journal of Energy Chemistry* *81* (2023): 583–592, https://doi.org/10.1016/j.jechem.2023.03.007.

10. F. Shen, H. Du, H. Qin, et al., “Mediating Triple Ions Migration Behavior via a Fluorinated Separator Interface toward Highly Reversible Aqueous Zn Batteries,” *Small* *20*, no. 1 (2024): 2305119, https://doi.org/10.1002/smll.202305119.
